# Early Impact of the *Western Journal of Emergency Medicine* CDEM/CORD Special Issue in Educational Research & Practice

**DOI:** 10.5811/westjem.2019.10.44484

**Published:** 2019-12-09

**Authors:** Jeffrey N. Love, Sally A. Santen, David P. Way, Brendan W. Munzer, Chris Merritt, Douglas S. Ander, John W. Cyrus

**Affiliations:** * George Washington University, Department of Emergency Medicine, Washington, District of Columbia; † Virginia Commonwealth University, Department of Emergency Medicine, Richmond, Virginia; ‡ Ohio State University, Department of Emergency Medicine, Columbus, Ohio; § University of Michigan, Department of Emergency Medicine, Ann Arbor, Michigan; ¶ Alpert Medical School of Brown University, Department of Emergency Medicine & Pediatrics, Providence, Rhode Island; || Emory University School of Medicine, Department of Emergency Medicine, Atlanta, Georgia; # Virginia Commonwealth University, Tompkins-McCaw Library for the Health Sciences, Richmond, Virginia

## Abstract

**Introduction:**

In 2015, with a stated goal of disseminating best teaching practices and developing a community of educational scholars, the Council of Emergency Medicine Directors (CORD) and the Clerkship Directors of Emergency Medicine (CDEM) created an annual Special Issue in Educational Research and Practice (Special Issue) in cooperation with the *Western Journal of Emergency Medicine*. The intention of this study was to analyze the impact of this effort to date.

**Methods:**

Bibliometric data was gathered on all four special issues, 2015–2019, from the Web of Science and then verified with the eScholarship website. Authorship, academic affiliation, date published, article type, and format were tabulated for descriptive analysis. Using metrics from Google Scholar, alternative scholarly impact metrics (altmetrics), and the eScholarship website, the authors identified top articles and grouped them into themes.

**Results:**

Of the 136 articles included in the first four years of the Special Issue, 126 represented peer-reviewed publications with an overall acceptance rate of 25.0% (126/505). Authors from this cohort represented 103 of the 182 (56.6%) Accreditation Council for Graduate Medical Education (ACGME) programs in existence at the time of the inaugural issue. Multi-institutional studies represented 34.9% (44/126) of the peer-reviewed publications. Traditional and alternative publication metrics are reported to assess the impact of articles from the Special Issues.

**Conclusion:**

The Special Issue is a proven outlet to share best practices, innovations, and research related to education. Additionally, the infrastructure of this process promotes the development of individual faculty and a community of teaching scholars.

## INTRODUCTION

Over the past decade, there has been a concerted effort on behalf of the Council of Emergency Medicine Directors (CORD) to increase the scholarly teaching provided by program faculty as well as to develop a community of practice related to education scholarship.^1^,^2^ Toward this end CORD, the Clerkship Directors of Emergency Medicine (CDEM), and the *Western Journal of Emergency Medicine* (*West*JEM) came together in 2015 to create an annual Special Issue in Educational Research and Practice.

The membership of CORD and CDEM represents the leadership and core educational faculty of emergency medicine (EM) training programs. Thus, this Special Issue was intended as a forum for best practices, innovations, and research related to education within the local and broader education communities.

Submissions are divided by topic into those most relevant to graduate medical education and undergraduate medical education with the former assigned to the CORD Editors and the latter to CDEM Editors. The Special Issue is generally published as the January issue of *West*JEM. As an open-access journal, *West*JEM provides the ability to publish as many articles as meet the editorial team’s standards. All articles meeting these standards are published online, while those deemed by the editors to be the most relevant to the EM education community and/or the best examples of education scholarship are also available in a 12–15 article print version. The purpose of this study was to profile the impact of the annual Special Issue on the target community since its first release in November 2015.

## METHODS

### Data Collection

In order to assess the impact of the first four CDEM/CORD Special Issues in Educational Research and Practice, we collected bibliometric data from a variety of sources. First, using Web of Science (WOS) (Clarivate Analytics, formerly Thomson Reuter’s), a librarian from the research team generated a list of all articles appearing in the 2015, 2017, 2018, and 2019 Special Issues. Data for each article were exported into a spreadsheet in February 2019, which included the following: author(s), article title, year of publication, affiliation, digital object identifier, and the times cited within the WOS Core Collection. We used the University of California eScholarship open-access web platform^3^ to verify and enhance the exported Web of Science data. Information about authors’ affiliations, article type, format of the article (print or online only), electronic publication date, number of institutions represented, and whether data was gathered from one or multiple institutions was abstracted and entered into the database.

Additional article metrics, such as the number of times that each article was cited, was exported from Scopus (Elsevier) and the web search engine Google Scholar (Mountain View, CA) to the spreadsheet. We obtained the Altmetric (London, England) scores from the *West*JEM website (https://westjem.com/), which reports this metric for each of its articles. Article page view and download data were obtained from the University of California eScholarship platform.

### Data Analysis

The Special Issues Guest Editors provided the overall submission volume and acceptance rates. The number of articles published in each of the following submission categories was also tabulated: commentary; educational scholarship insights; original research; education advances / innovations; and reviews. Authors’ institutional affiliation data exported from Web of Science was cleaned to collapse multiple names for one institution into a single identifier as listed in the roster of Accreditation Council for Graduate Medical Education (ACGME)-accredited EM training programs as of 2016. We used institutional identifiers to calculate the number of collaborative articles and multi-institutional studies. A multi-institutional study was defined a priori as a work that gathered data across more than one institution, either medical school or residency program.

From the four existing Special Issues, we identified separately the top 10 articles from each of the following impact indices for comparisons: times cited in Google Scholar, Altmetric score, and download count. These top articles were qualitatively coded independently by two authors (BWM and DA) to identify patterns or commonalities in topic between the most highly used articles. In cases where there was not agreement on the theme, a third author (SAS) served as an adjudicator. Coding was then reviewed by the authorship group.

## RESULTS

### Impact on Member Programs

From 2015 to 2019, the Special Issue published 136 articles. These consist of seven commentaries, three “educational scholarship insights,” 77 original research articles (67 full studies, 10 brief reports), 46 “educational advances” (31 full reports, 15 brief innovations), and three reviews. Of the 505 peer-reviewed articles submitted over this time period, 126 (25.0%) were accepted.

At the time the first Special Issue was published in November 2015, there were 182 ACGME-accredited EM training programs. The 528 unique authors of articles published represented 103 of these 182 accredited programs (56.6%). Nine additional authors came from EM programs accredited by the ACGME after 2016. The remaining authors represent non-ACGME approved EM programs from the United States (13), Europe (3), New Zealand (1), and Canada (1). Experience as lead author of a peer-reviewed publication in education was a requirement for selection as a reviewer. To date, 199 faculty have served as reviewers.

### Collaborative Efforts Based on Authorship

Of the 136 published works, 130 (95.6%) were collaborative efforts, and 69 (50.7%) had two or more authors from different ACGME-accredited EM programs. Multi-institutional data gathered by author(s) from a single ACGME institution (eg national surveys) represented 5.6% (7/126) and multi-institutional authors in 29.4% (37/126) of published peer-reviewed publications for a total of 34.9% (44/126).

### Published Article Performance

The top 10 articles based on Google Scholar citations, Altmetric scores, and the number of downloads are reported in Table 1. For Google Scholar citations, all but one of the articles were published in the 2015 issue. The “top 10 downloads” category has articles from 2015–2018. Finally, altmetrics have representation from all four years including three articles from 2019 within two months of their publication. Only four articles are found in the top 10 in more than one of these metrics. While all four are highly rated in downloads, two are also represented in Altmetric (Ferguson et al., 2017; Flanigan et al., 2015) and the remaining two in Google Scholar Citations (Gorgas et al., 2015; Wong et al., 2015) (Table 1).

**Figure 1:**
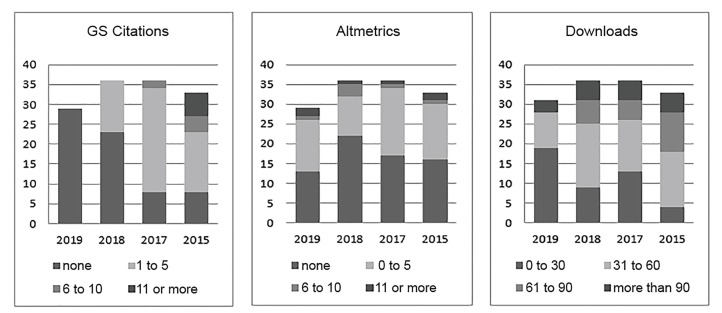
Distribution of performance by year of all *Western Journal of Emergency Medicine* Special Issues in Educational Research and Practice articles published based on Google Scholar citations, Altmetrics, and downloads. *GS*, Google Scholar.

Figure 1 shows the performance of all Special Issue articles published based on year and three metrics: Google Scholar citations; altmetrics; and downloads. Google Scholar citations appear to take time to develop and gradually increase. Altmetrics on the other hand demonstrate impact early on within weeks to months and remain relatively stable from that point on. Finally, the impact demonstrated by how many times an article has been downloaded can be seen within weeks to months of publication, but like citations, the impact tends to increase over time.

In comparing online articles also selected for the print version, it appears that they have no greater impact than those not selected based on top 10 performance in Google Scholar, altmetrics, and the number of downloads (Table 2).

In coding the topics of manuscripts in the top 10 based on Google Scholar citations, altmetrics, and number of downloads from 2015–2019, the designated study authors agreed on the categorization in 23/31 (74.2%). The remaining eight articles were adjudicated by a third author. Common themes identified across metrics included didactics, novel curricula, simulation, assessment/evaluation, scholarship, quality improvement/patient safety, recruitment/residency application, leadership, and clinical reasoning. No discernible difference between metric groups based on topic was found (Table 3).

## DISCUSSION

The purpose of this study was to describe the early impact of the first four editions of the Special Issue, with particular focus on the issue’s impact based on bibliometric data as well as its promotion of a culture of faculty development related to education scholarship. The CDEM and CORD leadership shared a goal of providing an education-focused Special Issue with early and lasting impact on education practice. The Special Issue was intended as a forum in which to share novel ideas, disseminate best education practices, and describe the findings of EM-based education research; early results demonstrate that the Special Issue has achieved its preliminary goals.

More than half of the articles published in the Special Issue represent original research, which perhaps may be a surprising outcome for a relatively young outlet. Acceptance rates over the first four editions of the Special Issue– at 25% – are in line with those reported by another journal early in its development,^4^ and reflect what the editors and CDEM/CORD leadership believe to be a rigorous review process, with acceptance of high-quality education scholarship. As an open-access journal, *West*JEM increases visibility and the likelihood of garnering medical attention and ultimately citation counts.^5^ Its open-access nature also allows the acceptance for online publication of all submissions that meet the editorial standards of the Special Issue. It also allows the editors to select articles for the print version and more prominent display, although this “special status” does not reflect the likelihood that an article will be more likely to achieve significant impact based on any given bibliometric measure.

A second goal of the *West*JEM Special Issue is to further develop and strengthen a community of education scholars in EM. This has been achieved in a number of ways. First, the Special Issue publishes a regular series of commentaries and “education scholarship insights” oriented toward professional development in education scholarship, highlighting existing controversies, areas of active investigation, and perspective of education scholars. Second, the Special Issue’s editors have assembled a cadre of associate guest editors and peer reviewers from across the EM education spectrum. These experiences are believed to be an important source of professional development in education scholarship for those who participate.^6^ Perhaps most importantly, the editorial team is charged with the mission of providing formative feedback to authors through manuscript review that highlights best practices and potential avenues for improvement, thereby providing professional development for budding education scholars.^7^

With 95% of all publications representing collaboration between two or more authors and 50.7% having representation from two or more ACGME-accredited programs, the Special Issue provides a platform for a collaborative scholarly collective further facilitating the professional development of education scholars. As important, multi-institutional research tends to demonstrate greater rigor than single-site studies, and are more likely to be generalizable to the education community as a whole.^8^ The representation of multi-institutional collaboration between authors and multi-site collection of research data presented in the Special Issue is a particular strength of the journal’s output in education scholarship to date (34.9% of peer-reviewed publications). By encouraging and highlighting such work, the Special Issue appears to be building a connection between educators and scholars, with the goal of supporting the development of a culture of educational practice that is built upon scholarship.^9^

Traditionally, a publication’s success has been based on the number of citations it receives measuring “intellectual impact.” High-impact papers generally reflect areas of development and intellectual interest at the time of the publication. Consistent with this concept, the top 10 Special Issue articles based on citations from 2015–2019 include such topics as the competency assessment, ultrasound in the emergency department, flipped classrooms, and emotional intelligence (Table 1).

There are three primary sources for traditional citation metrics: WOS; Scopus (Elsevier); and Google Scholar. Although each uses somewhat different databases, Google Scholar differs the most for its additional use of nontraditional, less academically rigorous sources such as conference proceedings, international non-English journals, course syllabi, blogs, and magazine articles.^10^–^12^ Google Scholar is unique in being freely accessible to individuals, while the others have associated costs as proprietary offerings. Prior work has shown traditional metrics based on citations takes two to five years to provide a sense of a paper’s impact that is intellectual in nature.^13^–^15^ With nine of the top 10 articles based on Google Scholar citations between 2015–2019 published in 2015, the findings of the current study aligns with this concept (Table 1).

In 2011, altmetrics was introduced providing a different perspective on impact.^16^ The altmetrics score represents a weighted approximation of the attention, which can be either good or bad, a publication receives based on various social media platforms such as Facebook, Twitter, blogs, Mendeley bookmarks, and Wikipedia.^17^ The most common input to this scoring is Twitter.^18^ For a number of reasons, not all possible sources are tracked, which is why a a question mark (?) and not a zero is placed in the Altmetric scoring circle if nothing is found. In 2015, Costas et al. reported that only 22.8% of health science research had an Altmetric score.^18^ In this study that number was 48.3%. It is generally agreed that altmetrics measures a different type of impact compared to traditional citations with counts reflecting interest in a topic from a broader community, including impact on government/policy institutions, educators, and the general public.^17^–^19^ As opposed to traditional metrics, altmetrics provides a measure of immediate impact seen within days (eg, Twitter) to months (eg, Mendeley) of publication.^14^,^19^,^20^ The data from the current study supports this assumption, as three of the top 10 altmetrics-measured articles are from the 2019 issue, two months after publication.

Prior work shows that there is a low but positive correlation between altmetrics scores and an article’s eventual citation count best predicted by the performance of “tag and save” resources such as Mendeley.^18^–^19^ Based on these differences from traditional metrics, one might assume that the articles demonstrating the most significant altmetrics impact would differ from those determined by traditional citations. This study found no measurable difference between the two (Table 3), although the small numbers generated may have limited our ability to determine a difference. It has been argued that altmetrics lack a sufficient validity argument based on rigorous evaluation to support its trustworthiness at the current time based on (1) lacking in underlying theory, (2) potential reporting bias, and (3) ease of gaming the system.^20^,^21^ The value and place of altmetrics and social media in determining impact continues to evolve.^12^,^22^,^23^

The third and final metric assessed by this study was collected from web-based platforms, reporting how often a published article is downloaded. Much like altmetrics, this impact is seen early after publication (ie, days to weeks) (Table 1). Several prior works have shown that this early measure of performance correlates with longer term citations counts and thus intellectual impact.^24^,^25^

## LIMITATIONS

This work represents a good faith effort to represent the impact of the Special Issue and the breadth of its reach into training programs in EM. Although this analysis documents the volume and type of educational research papers in the Special Issue, the analysis did not involve comparison to education research papers published in other journals during this or an earlier time frame. It is possible that the Special Issue merely diverted papers that would otherwise have been published elsewhere.

The decision to use the percentage of ACGME-accredited EM programs in 2016 as the denominator in calculations of program authorship representation was based on the large number of programs that have been accredited over the past four years, making the denominator a “moving target.” This is based in part on the inclusion of osteopathic graduate medical programs under the ACGME umbrella. This incoming group of programs represents an opportunity to broaden the Special Issue’s reach even further by promoting participation of these programs as a short-range goal.

## CONCLUSION AND FUTURE DIRECTIONS

The *West*JEM Special Issue in Educational Research and Practice, published annually over the past four years, has had significant impact both within and beyond the community of EM educators. The Special Issue has provided an outlet for education scholarship, discussion of current topics, debates in EM education, and dissemination of best practices. In addition, it has made significant strides in its stated goal of fostering collaboration across networks of educators and clinicians, while fostering the development of a community of practice in education scholarship.

The impact of the Special Issue is noted by its early outcomes as indicated by the altmetrics and download data presented –as well as forming the basis for future scholarship over time– as demonstrated by the citation data represented in the citation and more traditional impact data. Ongoing bibliometrics should be tracked to better understand and characterize the long-term impact of these papers in terms of citations and changes in educational practice.

## Figures and Tables

**Table 1:** Top 10 performing publications from the first four issues of the *Western Journal of Emergency Medicine* Special Issue in Educational Research and Practice (2015–2019) as determined by Google Scholar, Altmetrics, and downloads.

Rank	Title	Authors	Issue print/online (P/O)	GS citation
1	Does the Concept of the Flipped Classroom Extend to the Emergency Medicine Clinical Clerkship?	Heitz et al.	2015 (P)	30
2	Are Live Ultrasound Models Replaceable? Traditional versus Simulated Education Module for FAST Exam	Bentley et al.	2015 (P)	25
3	Emergency Medicine Residents Consistently Rate Themselves Higher than Attending Assessments on ACGME Milestones	Goldflam et al.	2015 (O)	14
3	Coordinating a Team Response to Behavioral Emergencies in the Emergency Department: A Simulation-Enhanced Interprofessional Curriculum	Wong et al.	2015 (P)	14
5	Teaching Emotional Intelligence: A Control Group Study of a Brief Educational Intervention for Emergency Medicine Residents	Gorgas et al.	2015 (O)	11
5	Model for Developing Educational Research Productivity: The Medical Education Research Group	Perry et al.	2015 (O)	11
5	Competency Assessment in Senior Emergency Medicine Residents for Core Ultrasound Skills	Schmidt et al.	2015 (O)	11
6	Efficient and Effective Use of Peer Teaching for Medical Student Simulation	House et al.	2017 (O)	10
6	Ultrasound Training in the Emergency Medicine Clerkship	Favot et al.	2015 (O)	10
6	What is the Prevalence and Success of Remediation of Emergency Medicine Residents?	Silverberg et al.	2015 (P)	10
				Altmetric score
1	Continuing Medical Education Speakers with High Evaluation Scores Use more Image-based Slides	Ferguson et al.	2017 (O)	47
2	Getting Published in Medical Education: Overcoming Barriers to Scholarly Production	Gottlieb et al.	2018 (P)	36
3	Teaching and Assessing ED Handoffs: A Qualitative Study Exploring Resident, Attending, and Nurse Perceptions	Flanigan et al.	2015 (P)	18
4	Morbidity and Mortality Conference in Emergency Medicine Residencies and the Culture of Safety	Aaronson et al.	2015 (P)	17
5	Standardized Video Interviews Do Not Correlate to United States Medical Licensing Examination Step 1 and Step 2 Scores	Egan et al.	2019 (O)	12
6	Recommendations from the Council of Emergency Medicine Residency Directors: Osteopathic Applicants.	Stobart-Gallagher et al.	2019 (O)	11
7	What Do They Want from Us? A Survey of EM Program Directors on EM Application Criteria	King et al.	2017 (O)	9
7	Tit-for-Tat Strategy for Increasing Medical Student Evaluation Response Rates	Malone et al.	2018 (P)	9
8	Bringing the Flipped Classroom to Day 1: A Novel Didactic Curriculum for Emergency Medicine Intern Orientation	Barrie et al.	2018 (O)	8
8	Free Open Access Medical Education (FOAM) Resources in a Team-Based Learning Educational Series	Fallon et al.	2018 (O)	8
8	Show Me the Money: Successfully Obtaining Grant Funding in Medical Education.	Gottlieb et al.	2019 (O)	8
				Times downloaded
1	Continuing Medical Education Speakers with High Evaluation Scores Use more Image-based Slides.	Ferguson et al.	2017 (O)	652
2	Coordinating a Team Response to Behavioral Emergencies in the Emergency Department: A Simulation-Enhanced Interprofessional Curriculum	Wong et al.	2015 (P)	544
3	A Randomized Trial of SMART Goal Enhanced Debriefing after Simulation to Promote Educational Actions	Aghera et al.	2018 (O)	228
4	Teaching Emotional Intelligence: A Control Group Study of a Brief Educational Intervention for Emergency Medicine Residents	Gorgas et al.	2015 (O)	224
5	Novel Airway Training Tool that Simulates Vomiting: Suction-Assisted Laryngoscopy Assisted Decontamination (SALAD) System	DuCanto et al.	2017 (O)	208
6	Characteristics of Real-Time, Non-Critical Incident Debriefing Practices in the Emergency Department	Nadir et al.	2017 (O)	205
7	Replacing Lectures with Small Groups: The Impact of Flipping the Residency Conference Day	King et al.	2018 (P)	145
8	Creating a Vision for Education Leadership	Martin et al.	2018 (O)	141
9	Clinical Reasoning: Defining It, Teaching It, Assessing It, Studying It	Gruppen	2017 (P)	140
10	Teaching and Assessing ED Handoffs: A Qualitative Study Exploring Resident, Attending, and Nurse Perceptions	Flanigan et al.	2015 (P)	138

*GS*, Google Scholar.

**Table 2:** Performance of those *Western Journal of Emergency Medicine* Special Issue published articles selected for print version in addition to online publication vs those published online only from 2015–2019.

	Google Scholar	Altmetrics	Downloads	Overall	% of total for group
Print & online	4	4	4	12	23.1% (12/54)
Online only	6	7	6	19	23.2% (19/82)

Percentage calculated by dividing the representation in the top 10 by the total number of articles published in that format.

**Table 3:** Topics of the *Western Journal of Emergency Medicine* Special Issue top 10 articles from 2015–2019.

Theme	Google scholar	Altmetric	Downloads	Overall (without repeat)
Didactics	1	4	2	6
Simulation	2	0	3	4
Assessment/evaluation	3	1	0	4
Novel curricula	3	0	1	3
Scholarship	1	2	0	3
Quality improvment/patient safety	0	1	2	2
Leadership	0	0	1	1
Clinical reasoning	0	0	1	1
Recruitment/residency application	0	3	0	3
Total				27
